# The relation of ApoE and COMT gene–gene interactions to cognitive and motor function in community-dwelling older adults: a pilot study

**DOI:** 10.3389/fnagi.2023.1206473

**Published:** 2023-08-31

**Authors:** Kendra L. Pizzonia, Julie A. Suhr, Leatha A. Clark, Brian C. Clark

**Affiliations:** ^1^Ohio Musculoskeletal and Neurological Institute (OMNI), Ohio University, Athens, OH, United States; ^2^Department of Psychology, Ohio University, Athens, OH, United States; ^3^Department of Biomedical Sciences, Ohio University Heritage College of Osteopathic Medicine, Athens, OH, United States

**Keywords:** apolipoprotein E4, COMT, cognition, motor functioning, aging, genotype

## Abstract

**Introduction:**

Certain genes increase the risk of age-related neurological dysfunction and/or disease. For instance, ApoE is a well-known gene carrying risk for Alzheimer’s disease, while COMT has been associated with age-related reductions in motor function. There is growing interest in the interrelationship between age-related changes in cognitive and motor function, and examining gene–gene interactions in this context. In this pilot study we examined the relations of the ApoE and COMT genes and their interaction to both cognitive and motor performance in community-dwelling older adults.

**Methods:**

We leveraged an archived dataset from a prior study on age-related muscle weakness in community-dwelling older adults. Sample size was between 72 and 82 individuals based on missing data. We examined the relationship of ApoE (Ɛ4 presence/absence), rs4680 SNP on the COMT gene (Val/Met, Val/Val, Met/Met), and sex on (1) overall cognitive functioning and specific cognitive domains known to decline in aging (processing speed, immediate and delayed memory, semantic and phonemic fluency, and executive functioning), and (2) indices of motor function (four square step test, short physical performance battery, grip strength/forearm lean mass, and purdue pegboard test).

**Results:**

Homozygous COMT genotypes were associated with worse global cognitive performance, immediate memory, and semantic fluency, but only for older adults with at least one ApoE Ɛ4 allele. There were main effects for COMT for delayed memory and a main effect for both COMT and ApoE for coding and phonemic fluency. Women scored higher than men in overall cognition, immediate and delayed memory, and semantic fluency. There were no main effects or gene interactions for a measure of executive functioning (trial making test part B) or any of the measures of motor function.

**Discussion:**

COMT, ApoE, and their interaction influence cognitive performance, but not motor functioning, in community dwelling older adults. Our work supports prior literature concluding that a heterozygous COMT genotype may be beneficial to sustain healthy cognitive functioning with advancing age for those who have a higher ApoE genetic risk status (at least one Ɛ4 allele). Future research should investigate interactions between COMT and ApoE in larger samples with comprehensive assessment of cognition and motor functioning.

## Introduction

It is well known that certain genes increase the risk of age-related neurological dysfunction and/or disease (Bedlack et al., 2000; Tang et al., 2019; Melzer et al., 2020). For instance, apolipoprotein E (ApoE) is involved in metabolism of lipoproteins and cholesterol redistribution, with the Ɛ4 allele conferring increased risk of cerebrovascular dysfunction (Yamazaki et al., 2019). ApoE is the strongest genetic predictor of Alzheimer’s disease (AD; Kunkle et al., 2019) and may enhance risk for mild cognitive impairment (MCI) and other dementias (Serrano-Pozo et al., 2021). However, there are conflicting findings about the effect of ApoE on cognition in healthy adults, with ApoE Ɛ4 associated with better cognitive functioning at midlife but higher rate of cognitive decline in older age (Gharbi-Meliani et al., 2021). While ApoE is often associated with memory, it is also related to executive functioning (Wolk et al., 2010; Luck et al., 2015). Moreover, ApoE alleles have been reported to influence the progression and incidence of a variety of peripheral nervous system diseases (Bedlack et al., 2000), and some studies (Petersen et al., 2016; Skoog et al., 2016), but not all (Alfred et al., 2014; Maltais et al., 2019; Kuo et al., 2020), have suggested that ApoE status is associated with age-related changes in motor function (e.g., grip strength). Observations of both cognitive and motor declines associated with genetic variability are consistent with the common cause hypothesis, which links age-related deterioration of brain function to age-related changes in both sensory-motor ability and cognitive functioning (Christensen et al., 2001).

Another gene related to both cognitive and motor function is Catechol-O-methyl transferase (COMT), with the rs4680 SNP on the COMT gene accounting for the greatest variance in cognitive function (Nackley et al., 2006) as well as motor function (Metti et al., 2017; Hupfeld et al., 2018; Moskowitz et al., 2021). COMT influences dopamine functioning, with the SNP resulting in either methionine (Met) or valine (Val) at position 158 creating Val/Val, Met/Met, or Val/Met allele combinations (Bäckman et al., 2006). According to a meta-analysis, COMT’s relationship to cognition has been primarily studied with psychiatric patients and younger samples, with few relationships between COMT and cognition identified (Barnett et al., 2008). One exception to this conclusion was on one working memory task (n-back) wherein individuals with at least one Val allele had higher accuracy, with a higher effect size estimate for older samples and also for samples with more women (Barnett et al., 2008). COMT as a risk factor of MCI or AD is not well understood (Pan et al., 2021), although some research has concluded that having at least one COMT Val allele is associated with cognitive decline in aging (e.g., Dixon et al., 2014). Further, COMT alleles have been associated with specific cerebrospinal fluid (CSF) biomarkers of AD, with lower CSF amyloid-β_42_ in Val/Val carriers but higher *t*-tau and *p*-tau_181_ in Met/Met carriers compared to Val/Met, suggesting that those with heterozygous alleles may have protection against AD (Babić Leko et al., 2020). Additionally, we have previously shown that older adults with the Val/Met intermediate COMT genotype demonstrated greater physical function/mobility than their homozygous counterparts (Moskowitz et al., 2021). Collectively, these findings support the inverted-u hypothesis of dopamine functioning (Goldman-Rakic et al., 2000; Goto et al., 2007). While these studies may also suggest support for the common cause hypothesis, existing studies do not tend to include both cognitive and motor tests in the same design.

Conceptually, it is plausible that COMT and ApoE interact to confer risk for age-related cognitive and motor dysfunction. While relatively little work has examined these gene–gene interactions as it relates to age-related changes in cognitive function, and to our knowledge no studies have examined this relationship in the context of motor function, there is observational evidence supporting our assertion. For instance, in a multi-site study of older adults, COMT was not an *independent* risk factor for MCI or AD, but there was a synergistic effect between COMT and ApoE Ɛ4 carriers (Martínez et al., 2009). Additionally, using data from the Victoria Longitudinal Study, Sapkota et al. (2017) found a moderating effect of ApoE, such that risk for executive function decline over 9 years was related to additive effects of COMT (and brain derived neurotrophic factor), but only in the presence of the ApoE Ɛ4 allele. These findings suggest further examination of ApoE and COMT interactions on both cognitive and motor function in the context of aging should be performed.

Accordingly, we explored the relations of ApoE, COMT, and their interaction to both clinically relevant cognitive task performance and motor function in community-dwelling older adults. To stimulate further work in this area, we leveraged an archived dataset from our prior work on age-related muscle weakness in community-dwelling older adults for this preliminary analysis (Wages et al., 2020; Moskowitz et al., 2021).

## Method

### Participants

Participants were from a deidentified archival database from a study on mechanisms of age-related muscle weakness in community-dwelling older adults (*n* = 89; UNCODE Study protocol registration NCT02505529). To qualify for the larger study, participants could not have known overt neuromuscular or neurological conditions that would influence motor functioning (e.g., Parkinson’s disease, multiple sclerosis, ALS, etc.), any ADL disability, or self-reported severe visual impairment. Participants had to have a body mass index between 18.0 and 40.0 kg/m^2^. A complete list of exclusion criteria can be found in our prior work (Wages et al., 2020; Moskowitz et al., 2021; Russ et al., 2022). See Table 1 for participant characteristics.

**Table 1:** Participant characteristics by measure.

Measure	*N*	Measure M(SD)	Age M(SD)	Sex (% women)	Race (% white)	ApoE Ɛ4 (% present)	COMT (%)		
							Val/Met	Val/Val	Met/Met
Cognitive abilities									
Overall cognition	82	106.60 (12.30)	72.61 (6.71)	66%	96%	26%	50%	27%	23%
Immediate memory	82	103.26 (13.29)	72.61 (6.71)	66%	96%	26%	50%	27%	23%
Delayed memory	82	101.74 (15.92)	72.61 (6.71)	66%	96%	26%	50%	27%	23%
Semantic fluency	82	10.33 (3.24)	72.61 (6.71)	66%	96%	26%	50%	27%	23%
Coding	82	10.85 (3.35)	72.61 (6.71)	66%	96%	26%	50%	27%	23%
Phonemic fluency	72	11.61 (2.19)	73.03 (4.99)	65%	96%	28%	53%	26%	19%
TMT B	72	12.18 (2.47)	73.03 (4.99)	65%	96%	28%	53%	26%	19%
Motor functioning									
FFST	78	9.68 (3.73)	74.69 (6.61)	67%	96%	24%	49%	28%	23%
SPPB	81	11.01 (1.31)	74.63 (6.75)	65%	96%	26%	49%	27%	24%
Normalized GS	79	30.40 (8.90)	74.77 (6.77)	65%	96%	24%	48%	28%	24%
PPBT	79	70.72 (16.22)	74.63 (6.59)	66%	96%	24%	49%	28%	23%

TMT B, Trail Making Test B (secs); FSST, Four square step test (secs); SPPB, Short physical performance battery; Normalized GR, Grip strength (kg) divided by forearm lean mass (kg); PPBT, Purdue Pegboard Test (secs).

### Procedure

All variables of interest for the present study were completed at visit 1 of the larger study, lasting 3 h. A licensed physical therapist conducted an interview to assess inclusion and exclusion criteria. Those who qualified then completed various questionnaires not included here, followed by cognitive testing using the Repeatable Battery for the Assessment of Neuropsychological Status (RBANS; Randolph, 2012), phonemic fluency (FAS; Strauss et al., 2006), and the Trail Making Test (Strauss et al., 2006). Additionally, participants performed the four square step test (FSST), short physical performance battery (SPPB), grip strength, and purdue pegboard test (PPBT) as measures of motor function as per our prior description (Wages et al., 2020; Russ et al., 2022). Participants also underwent a dual-energy x-ray absorptiometry scan to quantify lean mass, which was subsequently used for grip strength normalization (discussed below in further detail). Participants provided a buccal swab for genetic testing and then completed physical functioning, electrophysiology, neuroimaging, and nutrition assessments not included here.

### Measures

#### Genomic analysis

Participants’ buccal swabs were obtained with an Isohelix SK1 buccal swabs (Isohelix DNA Sampling and Purification, Unit 2 Roebuck Business Park, Ashford Road, Harrietsham, Kent, UK; Cat. #SK-1S), and stored with an Isohelix Dri-Capsules (Cat. #SGC-50) at the time of collection. DNA extraction and genetic analyses were conducted at Ohio State University’s Comprehensive Cancer Center Genomics Shared Resource facility. DNA was isolated using Maxwell® RSC Automated Nucleic Acid Extraction instrument (Promega Corporation, 2,800 Woods Hollow Road Madison, WI; Cat. #AS4500) and Maxwell® RSC Buccal Swab DNA Kit (Cat. #AS1640) as we have previously described (Moskowitz et al., 2021).

#### Assessment of cognitive function

##### Repeatable battery for the assessment of neuropsychological status

The RBANS is a cognitive screening measure assessing immediate memory, delayed memory, visuospatial skills, language, attention, and psychomotor processing speed (Randolph, 2012). We used the age-corrected total index score as a measure of overall cognition. To assess learning and memory, we used the age-corrected immediate memory and delayed memory index scores. We also used age-corrected scaled scores from semantic fluency (to assess semantic memory) and coding (to reflect processing speed; Randolph, 2012). Three participants were 90 years of age or older and were coded with 80–89-year-old norms.

##### Phonemic fluency

Phonemic fluency was assessed by asking participants to list words beginning with F, A, and S for one minute per letter to represent the executive functioning component of verbal fluency. Age-corrected scaled scores were used (Tombaugh et al., 1999).

##### Trial making test

The Trail Making Test is a paper-and-pencil speeded task that measures the length of time required to connect dots in order. TMT part B represented the executive function component of task-switching and age-corrected scaled scores were used (Steinberg et al., 2005).

### Assessment of motor function

#### The four square step test

We used the time to complete the FSST, a multidirectional stepping test, as an index of lower extremity motor function that includes an executive functioning component. This test heavily challenges motor planning and initiation, as well as motor sequencing and recall, and incorporates musculoskeletal, and likely, peripheral sensory factors (i.e., strength and joint range of motion of the hip and ankle, as well as sensation/proprioception; Dite and Temple, 2002; Jung and Yamasaki, 2016; Moore and Barker, 2017; Saito et al., 2019; Wages et al., 2020). The FSST was conducted as we have previously described (Wages et al., 2020). Briefly, participants were required to step in a predetermined sequence over four 76-cm-long pieces of white tape, placed in a cross configuration on the ground over dark colored carpet. Participants were instructed to complete the sequence as fast as possible without touching the pieces of white tape and time to task completion was recorded. The trial was considered a failure and repeated if the sequence was not completed correctly, the participant lost their balance, or if a foot touched the tape line. Trials were only repeated if the participant failed and each participant performed 3 trials with 30 s rest between trials. The times were subsequently averaged.

#### Short physical performance battery

The short physical performance battery (SPPB) was used as a composite index of lower extremity physical function (Guralnik et al., 1994). The SPPB assesses standing balance (side-by-side, semi-tandem, and tandem), 4-meter usual gait speed, and 5x-chair rise test time with the composite score ranging from zero to 12.

#### Grip strength normalized to forearm lean mass

Grip strength was assessed with a JAMAR® dynamometer; (Model 5,030 J1; Lafayette instrument Co.; Lafayette, IN, United States), adjusted to fit participants’ hands as described elsewhere (Wages et al., 2020). Three trials per arm were obtained and average grip strength calculated. Lean mass was assessed *via* dual-energy X-ray absorptiometry (DXA) (Hologic discovery QDR model Series, Waltham, MA, United States) (Wages et al., 2020). Forearm lean tissue mass was extracted from the whole-body scan (upper extremities distal to the elbow) using manufacturer’s software (Hologic APEX, Version 4.0.2). Grip strength was subsequently expressed relative to forearm lean tissue mass, which controls for between subject differences in lean mass and will conceptually be more reflective of neurological mechanisms of grip strength. It should be noted that while DXA-derived measures of lean mass are not sensitive to *change* in muscle mass, that it does serve as a reasonable proxy for cross-sectional studies (*r* = 0.89 when compared to MRI-derived muscle volume; Tavoian et al., 2019).

#### Purdue pegboard test

Manual dexterity was tested with a modified purdue pegboard test (PPBT; Model 32,020; Lafayette instrument Co., Lafayette, Indiana), which has been found to have good-to-excellent reliability in older adults (Desrosiers et al., 1995). Participants completed two trials per hand in which they filled 25 holes on the ipsilateral side with pegs, as fast as possible. Average time per hand was calculated, and then averaged across the dominant and non-dominant side.

### Statistical analyses

ApoE status was defined as presence/absence of Ɛ4 and the rs4680 SNP on COMT was classified into Val/Met, Val/Val, and Met/Met allele combinations. Two (ApoE) by three (COMT) by two (self-reported sex) ANOVAs were conducted to investigate how ApoE, COMT, self-reported sex, and their interactions were related to various dependent variables (e.g., cognitive domains, motor functioning). A pre-set alpha level of 0.05 was required for statistical significance with effect size characterized by partial eta squared (η^2^_p_). Due to sample size limitations and the exploratory nature of our analyses, we did not correct for multiple comparisons. Pearson correlations were used to establish preliminary relationships between our cognitive and motor functioning measures. Biological sex assigned at birth was self-reported and included as a binary term (man/woman).

## Results

### Initial relationships between cognitive and motor functioning

We found that faster FSST performance was associated with a higher scaled score for processing speed (Coding, *r* = −0.336, *p* = 0.003) and two measures of executive functioning (TMT B, *r* = −0.280, *p* = 0.020; phonemic fluency, *r* = −0.255, *p* = 0.035). Better physical performance as measured by SPPB was related to a higher scaled score for processing speed (Coding, *r* = 0.302, *p* = 0.006) and one measure of executive functioning (TMT B, *r* = 0.239, *p* = 0.044). Higher normalized grip strength was related to a higher scaled score for processing speed (Coding, *r = 0.*377, *p* < 0.001). Faster PPBT performance was related to a higher scaled score for semantic fluency (*r* = −0.226*, p* = 0.045). See Table 2 for a complete correlation table.

**Table 2:** Pearson correlations between cognitive tasks and motor functioning measures.

	Overall index	Immediate memory	Delayed memory	Semantic fluency	Coding	Phonemic fluency	TMT B	FSST	SPPB	Grip strength
Immediate memory	0.616***	–								
Delayed memory	0.718***	0.551***	–							
Semantic fluency	0.429***	0.147	0.211	–						
Coding	0.463***	0.073	0.132	0.249*	–					
Phonemic fluency	0.434***	0.144	−0.003	0.312**	0.389***	–				
TMT B	0.411***	0.236*	0.111	0.181	0.382***	0.287*	–			
FSST	−0.159	−0.093	0.028	−0.062	−0.336**	−0.255*	−0.280*	–		
SPPB	0.102	0.021	0.007	0.028	0.302**	0.094	0.239*	−0.713***	–	
Normalized GS	0.151	0.102	−0.085	0.147	0.377***	0.197	0.229	−0.375***	0.338***	–
PPBT	−0.180	−0.011	−0.053	−0.226*	−0.141	−0.021	−0.144	0.588***	−0.367***	−0.412***

Significant correlations were denoted as follows: *p < 0.05, **p < 0.01, ***p < 0.001.

TMT B, Trail Making Test B (secs); FSST: Four square step test (secs); SPPB, Short physical performance battery; Normalized GR, Grip strength (kg) divided by forearm lean mass (kg); PPBT, Purdue Pegboard Test (secs).

### Overall cognition

**Figure 1:**
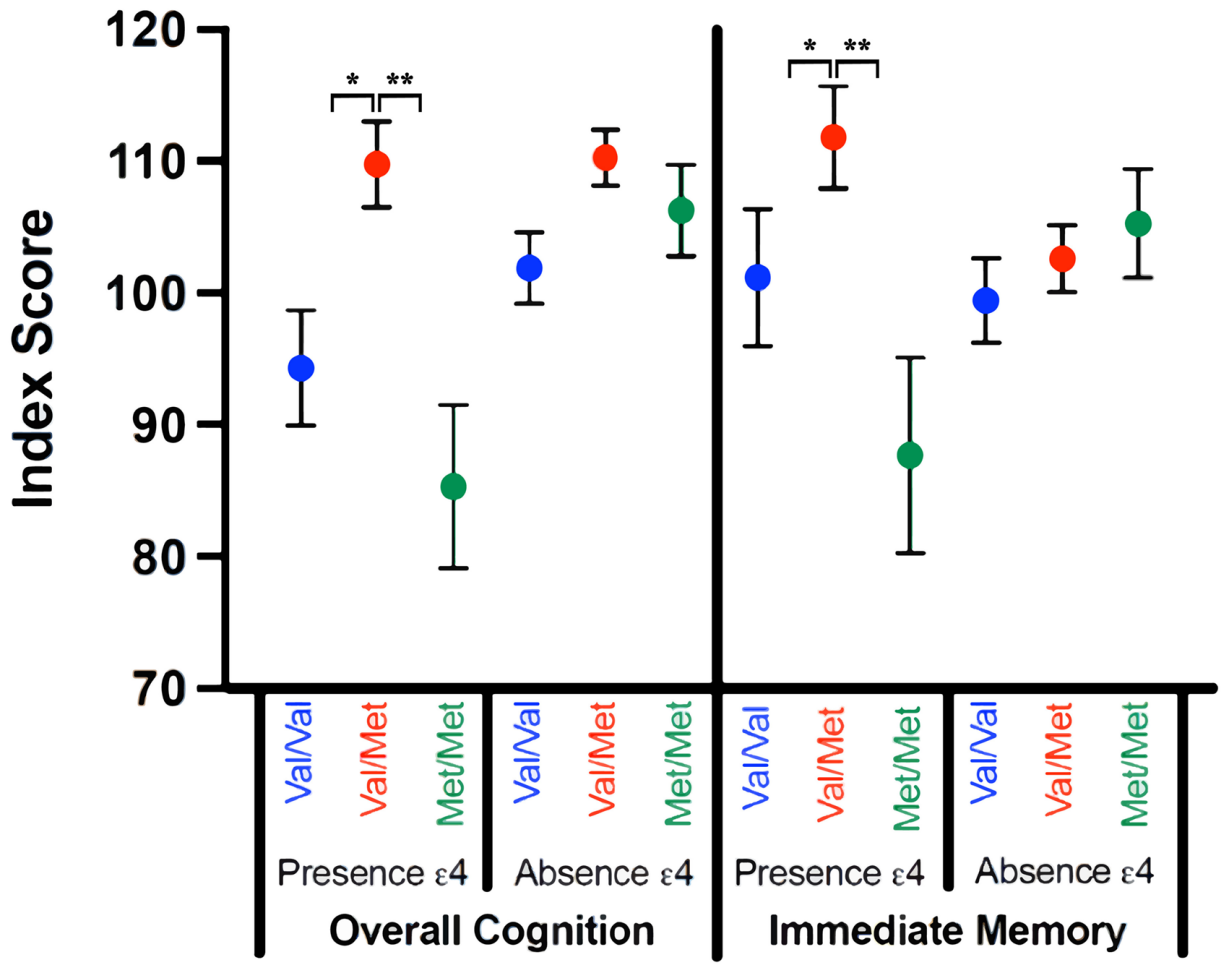
Significant Gene–Gene Interactions by RBANS Index Score. Index scores for overall cognitive performance and immediate memory on the RBANS are compared for those with (presence Ɛ4) and without (absence Ɛ4) an ApoE Ɛ4 allele across the COMT SNPs status (Val/Val; Val/Met; Met/Met). Significant effects were denoted as follows: *p < 0.05, **p < 0.01, ***p < 0.001.

Women scored higher than men, *F*(1,70) = 8.754, *p* = 0.004, η^2^_p_ = 0.111; there were no interactions between self-reported sex and genetic factors (*p*s = 0.160–0.926). There was a significant gene–gene interaction, *F*(2,70) = 3.230, *p* = 0.046, η^2^_p_ = 0.084. For participants with no Ɛ4, COMT was not a significant factor, *F*(2,61) = 3.026, *p* = 0.057, η^2^_p_ = 0.099. However, in those with at least one Ɛ4 allele, there was a significant effect of COMT, *F*(2,21) = 7.114, *p* = 0.007, η^2^_p_ = 0.487, such that individuals with Val/Met scored significantly higher than individuals with either Val/Val (*p* = 0.017) or Met/Met (*p* = 0.005), with no difference between Val/Val and Met/Met phenotypes (*p* = 0.280; See Figure 1).

### Immediate memory

Women scored higher than men, *F*(1,70) = 5.163, *p* = 0.026, η^2^_p_ = 0.069; there were no interactions between self-reported sex and genetic variables (*p*s = 0.061–0.948). There was a significant gene interaction, *F*(2,70) = 3.871, *p* = 0.025, η^2^_p_ = 0.100. There was no main effect for COMT in those with no Ɛ4, *F*(2,61) = 0.594, *p* = 0.555, η^2^_p_ = 0.021. For those with at least one Ɛ4 allele, there was an effect of COMT, *F*(2,21) = 7.164, *p* = 0.007, η^2^_p_ = 0.489, following the same pattern as overall cognition; individuals with Val/Met scored significantly better than those with either Val/Val (*p* = 0.043) or Met/Met (*p* = 0.003), with no difference between Val/Val and Met/Met phenotypes (*p* = 0.110).

### Delayed memory

Women had better delayed memory, *F*(1,70) = 11.950, *p* = 0.001, η^2^_p_ = 0.146; there were no interactions between self-reported sex and genetic variables (*p*s = 0.152–0.845). There was no main effect of ApoE, *F*(1,70) = 2.671, *p* = 0.107, η^2^_p_ = 0.037, and no gene interaction (*p* = 0.798, η^2^_p_ = 0.006). There was a main effect of COMT, *F*(2,70) = 3.580, *p* = 0.033, η^2^_p_ = 0.093, with individuals with Val/Met scoring higher than those with Met/Met (*p* = 0.013; See Figure 1).

### Semantic memory

**Figure 2:**
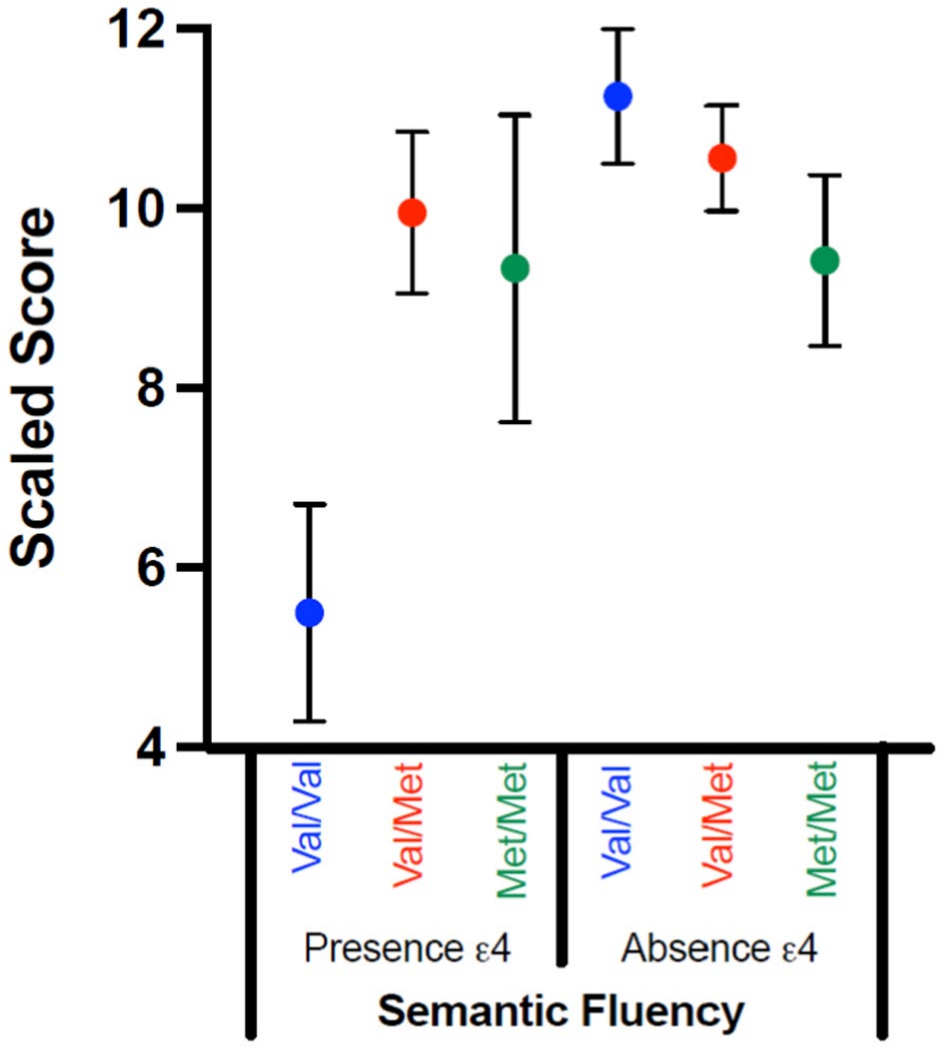
Significant Gene–Gene Interactions by Scaled Scores. Scaled scores for Scaled Fluency on the RBANS are compared for those with (presence Ɛ4) and without (absence Ɛ4) an ApoE Ɛ4 allele across the COMT SNPs status (Val/Val; Val/Met; Met/Met). Although the interaction was significant, none of the post-hoc comparisons were significant; this interaction appears to be driven by the Val/Val vs. Val/Met comparison for those with at least one Ɛ4 allele.

Women had better semantic fluency performance, *F*(1,70) = 4.759, *p* = 0.033, η^2^_p_ = 0.064, with moderate effect size; self-reported sex did not interact with genetic variables (*p*s = 0.307–0.688). There was a significant gene interaction, with moderate-to-large effect size, *F*(2,70) = 4.790, *p* = 0.011, η^2^_p_ = 0.120. When the interaction was examined, COMT was not significant in either the absence (*p* = 0.295; η^2^_p_ = 0.043) or presence (*p* = 0.061; η^2^_p_ = 0.311) of Ɛ4. That said, the significant interaction appears to be driven by the Val/Val vs. Val/Met comparison for individuals who had at least one Ɛ4 allele, with individuals with Val/Met having higher scores than those with Val/Val (*p* = 0.022; Figure 2).

### Processing speed

There was no self-reported sex difference (*p* = 0.190, η^2^_p_ = 0.024) or self-reported sex by gene interactions (*p*s = 0.337–0.811). Individuals with at least one Ɛ4 allele obtained lower scores on Coding, *F*(1,70) = 10.257, *p* = 0.002, η^2^_p_ = 0.128. There was a main effect of COMT, *F*(2,70) = 3.245, *p* = 0.045, η^2^_p_ = 0.085, with individuals with Val/Met scoring higher than those with Met/Met (*p* = 0.027). There was no gene–gene interaction (*p* = 0.162, η^2^_p_ = 0.051).

### Executive functioning

#### Phonemic fluency

There was no self-reported sex difference (*p* = 0.513, η^2^_p_ = 0.007) and no self-reported sex by gene interactions (*p*s = 0.148–0.418). Individuals with an Ɛ4 allele had lower performance than those without an Ɛ4 allele, *F*(1,61) = 5.282, *p* = 0.025, η^2^_p_ = 0.080. There was a main effect of COMT, *F*(1,61) = 3.437, *p* = 0.038, η^2^_p_ = 0.101, with individuals with Val/Met scoring higher than those with Val/Val (*p* = 0.025). There was no gene interaction (*p* = 0.060, η^2^_p_ = 0.088).

#### Trail making test

TMT part B was not significantly related to ApoE (*p* = 0.907, η^2^_p_ < 0.001), COMT (*p* = 0.806, η^2^_p_ = 0.007), or self-reported sex (*p* = 0.796, η^2^_p_ = 0.001). There were no significant interactions (*p*s = 0.374–0.779).

### Motor functioning

#### Four square step test

FSST was not significantly related to ApoE (*p* = 0.829, η^2^_p_ = 0.001), COMT (*p* = 0.271, η^2^_p_ = 0.038) or self-reported sex (*p* = 0.243, η^2^_p_ = 0.020). There were no significant interactions (*p*s = 0.237–0.687).

#### Short physical performance battery

SPPB was not significantly related to ApoE (*p* = 0.375, η^2^_p_ = 0.011), COMT (*p* = 0.293, η^2^_p_ = 0.035) or self-reported sex (*p* = 0.241, η^2^_p_ = 0.020). There were no significant interactions (*p*s = 0.093–0.807).

#### Grip strength normalized to forearm lean mass

Normalized grip strength was not significantly related to ApoE (*p* = 0.318, η^2^_p_ = 0.015), COMT (*p* = 0.650, η^2^_p_ = 0.013) or self-reported sex (*p* = 0.095, η^2^_p_ = 0.041). There were no significant interactions (*p*s = 0.297–0.745).

#### Purdue pegboard test

PPBT was not significantly related to ApoE (*p* = 0.411, η^2^_p_ = 0.010), COMT (*p* = 0.338, η^2^_p_ = 0.031) or self-reported sex (*p* = 0.425, η^2^_p_ = 0.009). There were no significant interactions (*p*s = 0.322–0.995).

## Discussion

We found preliminary evidence to suggest that the interaction between COMT and ApoE relates to performance in areas of cognition specifically associated with Alzheimer’s disease, including loss of episodic and semantic memory as represented by immediate learning/memory and semantic fluency (Butters et al., 1987; Vonk et al., 2020), as well as overall cognition. Specifically, only in the context of an ApoE Ɛ4 allele, individuals with Val/Met performed better in these cognitive domains. An exception to this pattern was that individuals with Val/Met performed better in delayed memory, with no moderation by Ɛ4 status. Theoretically, the inverted-u hypothesis of dopamine functioning indicates that having too much and too little dopamine could result in negative outcomes (Goldman-Rakic et al., 2000; Goto et al., 2007; Mier et al., 2010). This suggests that there may be a benefit for those with mixed genetic status, which is consistent with the pattern of our findings and with prior studies showing that Val/Val status is associated with worse cognitive outcomes (Martínez et al., 2009; Mier et al., 2010) and mixed COMT variants being related to relatively less accumulation of amyloid and tau (Babić Leko et al., 2020).

In other cognitive domains, main effects of both genes were observed, but no interaction between genes. In both processing speed (Coding) and phonemic fluency, those with at least one ApoE Ɛ4 allele performed worse, while those with COMT Val/Met alleles performed better. These findings support the well documented negative impact of ApoE Ɛ4 on cognitive functioning and are further support for the inverted-u hypothesis of dopamine.

Of note, although we did not find any interactions of gene variants with self-reported sex, our findings are generally consistent with a female advantage in certain cognitive abilities, even during aging (Hirnstein et al., 2023).

Interestingly, we did not find gene effects for one measure of executive functioning or for any of our motor functioning tasks. Overall, motor tasks had little to no association with cognitive measures typically associated with Alzheimer’s dementia (memory, semantic fluency) and for which we found gene–gene interactions. However, three of four motor tasks were significantly related to at least one psychomotor processing speed measure (Coding, Trail Making Test part B). This is consistent with research relating speeded motor tasks with executive functioning (Falck et al., 2017). However, this may also be due to a shared feature of psychomotor speed, as phonemic fluency, a speeded executive measure without a motor component, was only related to one measure of motor functioning. Of note, PPBT, which involves motor speed, was not related to measures of psychomotor processing speed measures.

There were several notable limitations in the present study, including sample size and a demographically homogenous sample (i.e., primarily white, well-educated, and community dwelling). Thus, our findings may not be generalizable to groups not represented in our sample and are preliminary. We were underpowered for our analyses, especially any three way interactions with self-reported sex, and, given prior work that ApoE and COMT interactions were associated with differential transition to MCI or AD based on sex (Martínez et al., 2009), future research should continue to examine sex differences in genetic factors on cognition with sufficiently powered samples. Of note, as in most prior research, we were not able to investigate biological factors underpinning self-reported sex differences. Additionally, studies showing differences in semantic fluency between men and women suggest this may be category dependent (Hirnstein et al., 2023); our study design limited our ability to explore this question, representing an area of future research. Given our small sample size, we were unable to combine other COMT SNPs into a haplotype. Another limitation of our dataset is that we did not collect education-related information, thus we are unable to consider the role education may have had on our findings. Further, our data was cross-sectional, and longitudinal studies are necessary to assess whether these genes are related to cognitive decline over time. Future studies should include COMT genotypes as a potential interaction in polygenetic risk scores with other genes thought to impact aging (e.g., BDNF) including a more diverse sample and should use such variables to predict cognitive decline over time.

## Conclusion

In summary, the common cause hypothesis in aging attributes changes in cognitive and motor functioning to the same neurobiological mechanisms. To date, there are no studies exploring how ApoE and COMT may interact to influence both motor functioning and cognition in older adults. Our findings suggest that interactions between ApoE and COMT are found in cognitive domains typically associated with Alzheimer’s disease and are less related to factors related to healthy cognitive and motoric aging broadly. Specifically, we found a benefit of heterozygous COMT genetic status, with Val/Met alleles associated with better performance in overall cognitive functioning, learning/memory, and semantic fluency for those with at least one ApoE Ɛ4 allele. These results are supported by the inverted-u hypothesis with a heterozygous COMT genotype, and thus an intermediate amount of dopamine activity, associated with better cognitive performance in domains described above. Together with other studies (Martínez et al., 2009; Dixon et al., 2014; Babić Leko et al., 2020), our work supports the inclusion of COMT into polygenetic risk scores for MCI and AD as it interacts with ApoE status for selective cognitive domains associated with these conditions. Additionally, by characterizing patients’ genetic status we can move toward developing individual risk management plans for older adults to sustain overall well-being.

## Data availability statement

The original contributions presented in the study are included in the article/supplementary materials, further inquiries can be directed to the corresponding author.

## Ethics statement

The studies involving humans were approved by Ohio University Institutional Review Board. The studies were conducted in accordance with the local legislation and institutional requirements. The participants provided their written informed consent to participate in this study.

## Author contributions

BC, LC, and JS made substantial contributions to the conception or design of the work. BC and LC were involved in the acquisition and analysis of data. KP and JS performed the statistical analysis. KP drafted the manuscript. BC, LC, and JS revised the manuscript for important intellectual content. All authors contributed to the article and approved the submitted version.

## Funding

This work was supported by the National Institute on Aging (grant numbers: R01AG044424 and R01AG067758).

## Conflict of interest

In the past 5-years, BC has received research funding from NMD Pharma, Regeneron Pharmaceuticals, Astellas Pharma Global Development, Inc., RTI Health Solutions, Myolex Inc., and OsteoDx, Inc. for contracted studies that involved aging related research. In the past 5-years, BC has received consulting fees from Regeneron Pharmaceuticals, Zev industries, and the Gerson Lehrman Group for consultation specific to aging-related research. BC is a co-founder with equity of OsteoDx, Inc.

The remaining authors declare that the research was conducted in the absence of any commercial or financial relationships that could be construed as a potential conflict of interest.

## Publisher’s note

All claims expressed in this article are solely those of the authors and do not necessarily represent those of their affiliated organizations, or those of the publisher, the editors and the reviewers. Any product that may be evaluated in this article, or claim that may be made by its manufacturer, is not guaranteed or endorsed by the publisher.
